# JAtlasView: a Java atlas-viewer for browsing biomedical 3D images and atlases

**DOI:** 10.1186/1471-2105-6-47

**Published:** 2005-03-09

**Authors:** Guangjie Feng, Nick Burton, Bill Hill, Duncan Davidson, Janet Kerwin, Mark Scott, Susan Lindsay, Richard Baldock

**Affiliations:** 1 MRC Human Genetics Unit, Western General Hospital, Crewe Road, EH4 2XU, Edinburgh, UK; 2 The Institute of Human Genetics, University of Newcastle, International Centre for Life, Central Parkway, Newcastle-upon-Tyne, NE1 3BZ, UK

## Abstract

**Background:**

Many three-dimensional (3D) images are routinely collected in biomedical research and a number of digital atlases with associated anatomical and other information have been published. A number of tools are available for viewing this data ranging from commercial visualization packages to freely available, typically system architecture dependent, solutions. Here we discuss an atlas viewer implemented to run on any workstation using the architecture neutral Java programming language.

**Results:**

We report the development of a freely available Java based viewer for 3D image data, descibe the structure and functionality of the viewer and how automated tools can be developed to manage the Java Native Interface code. The viewer allows arbitrary re-sectioning of the data and interactive browsing through the volume. With appropriately formatted data, for example as provided for the Electronic Atlas of the Developing Human Brain, a 3D surface view and anatomical browsing is available. The interface is developed in Java with Java3D providing the 3D rendering. For efficiency the image data is manipulated using the Woolz image-processing library provided as a dynamically linked module for each machine architecture.

**Conclusion:**

We conclude that Java provides an appropriate environment for efficient development of these tools and techniques exist to allow computationally efficient image-processing libraries to be integrated relatively easily.

## Background

Three-dimensional (3D) images are now commonplace in biomedical research. Techniques for direct capture of 3D data are widespread and new techniques are becoming available, [1,2] to complement existing sectioning methods [3], confocal and micro-CT/MRI [4]. In addition such data is being stored in databases that can be accessed freely (EADHB[5], EMAP[6], BIOIMAGE[7], and MRIMA[8]) and many more such atlases and bioinformatics resources will become available. There are a number of tools available for browsing such data, but they are either commercial with a significant cost for the user (e.g. AVS/Express, VolRen, Amira, Analyse) or free but tied to a specific architecture. Systems based purely on an architecture neutral language such as Java (e.g. ImageJ[9]) can be slow when processing large 3D volume images and have not been developed with the 3D atlas browsing application in mind. The purpose of this work is to combine the machine-architecture independence of Java, with a highly portable, freely available fast and efficient C-coded image processing library tuned to the requirements of the atlas browsing and data analysis task. The Java Atlas-Viewer (JAtlasView) interface has been developed as a series of modules that can be readily re-used within other applications to build more complex interfaces. The Java interface elements and the image processing library can be downloaded from the EADHB and EMAP web-sites. 3D images are regularly captured as part of biomedical research. In many fields the most useful and regularly used visualisation of the grey-level or colour voxel image is to view sections. These are 2D images generated by digitally cutting throught the volume and mimic the traditional mechanism of physical microtome sectioning for revealing detailed structure. The benefit of digital models is that the sectioning plane, orientation and position, can be selected arbitrarily to suit the required usage and the volume can be scanned interactively.

For the expert viewer, digital re-sectioning is sufficient for data-analysis but for others, panning through the volume at non-standard angles leads to disorientation. In addition if used in conjunction with atlas information in which the histology images are segmented in terms of the recognisable tissues, the building of a 3D view of the tissue/anatomical components is very difficult, particularly when learning the anatomy. This orientation and structural visualisation problem is solved by using 3D visualisation of the underlying tissue coupled with interactive feedback of the section location within the volume.

The basic structure of the JAtlasView is therefore a combination of a 3D feedback window with a number of section views. Each section view is independent and feedback is provided by displaying the position of the section within the 3D volume either as a simple polygon indicating the plane of section or as a full grey-level image, displayed appropriately in 3D. In addition each section will display the intersection with all other sections currently being viewed.

In this short note we describe the structure of the software and the functionality of the interface. This application is directed to the use of the EADHB and EMAP atlases and for browsing 3D grey-level data. In the first instance the data is formatted as a Woolz image structure [10], tools are available for data conversion and future versions will include this as standard.

## Implementation

The software design has been developed to meet a number of code requirements:

• portability to all major architectures – Unix/X11, Microsoft Windows and Macintosh,

• fast and efficient image processing, compatible with existing formats and interfaces,

• freely available code and modular design so display elements and functionality can be easily included in other applications and

• the user-interface should be mappable to the "look and feel" of the specific machine window system.

The portability and user-interface behaviour requirements are satisfied by using Java as the language and environment for the user interface level. For image processing we have adopted the ANSI standard C image processing library Woolz. This already includes the required functionality for calculating and manipulating section views through 3D voxel images and is open-source software.

A potential problem with Java is that it can be very inefficient for heavy numerical work (such as image processing) and the effort required to port existing libraries (for example Woolz is 185 K lines of code) to Java is too high. To solve this we use the tools within Java for accessing "native" code so that the computational work is undertaken in C. The management and coding of the interface is potentially time-consuming and prone to error with any small change in the C code requiring complementary effort to modify the native interface code. We have addressed this problem by implementing an automated method which will build the interface directly from the C-library header files. By adopting a standard convention for function prototyping it is possible to use a parser generator, javacc[11] to build a java program that can analyse the C-headers and automatically generate the Java class files and matching C-library files required for the Java native interface (JNI). This has made it possible to relegate generating the interface to an automatic process hidden from the primary code develoment, in fact without this development the system would be very difficult to manage.

Two other key choices have been made in the design of the code structure. The first is that the 3D visualisation and feedback should be developed at a level independent of the underlying hardware within an environment that allows a high level of abstraction of the 3D view. The java 3D extension to the core Java environment provides such a model and we have adopted this as standard. Java 3D is available for all Java 2 platforms.

The second key choice is that the software will be delivered using Java Webstart[12]. This is a freely available application that will download code across the internet and check system, version and supplementary module requirements. In addition it will start the application and maintain a local cached version. The local cache will be used for fast start if it is the same version as currently available at source or if the machine is off-line. Source code is maintained with *CVS*[13] for version management and tracking and GNU *gmake *for compilation. The interfaces are developed using Borland JBuilder[14] or a standard editor (vi) and documented using Javadoc/Doxygen[15].

Help is provided in two ways, the first is a simple popup "balloon" help on mouse-over and as a series of help files arranged using JavaHelp[16] which provides an indexing, search and context help facility. The help html files are generated using DreamWeaver [17] and maintained in a CVS repository.

Java is now widely used and the first choice for new applications that require portability across machine architectures. It is a strict object-oriented language and interfaces adhere to the *model-view-controller *(MVC) design pattern [18]. Java also defines a standard under the name *java-bean*, that components should meet to guarantee the MVC behaviour and enable easy re-use in other applications using CASE tools. We have adopted this standard for the JAtlasViewer application so that individual interface elements, e.g. the section panel or even our extended view of the slider, can be used simply and conveniently in other code.

## Results

**Figure 1:**
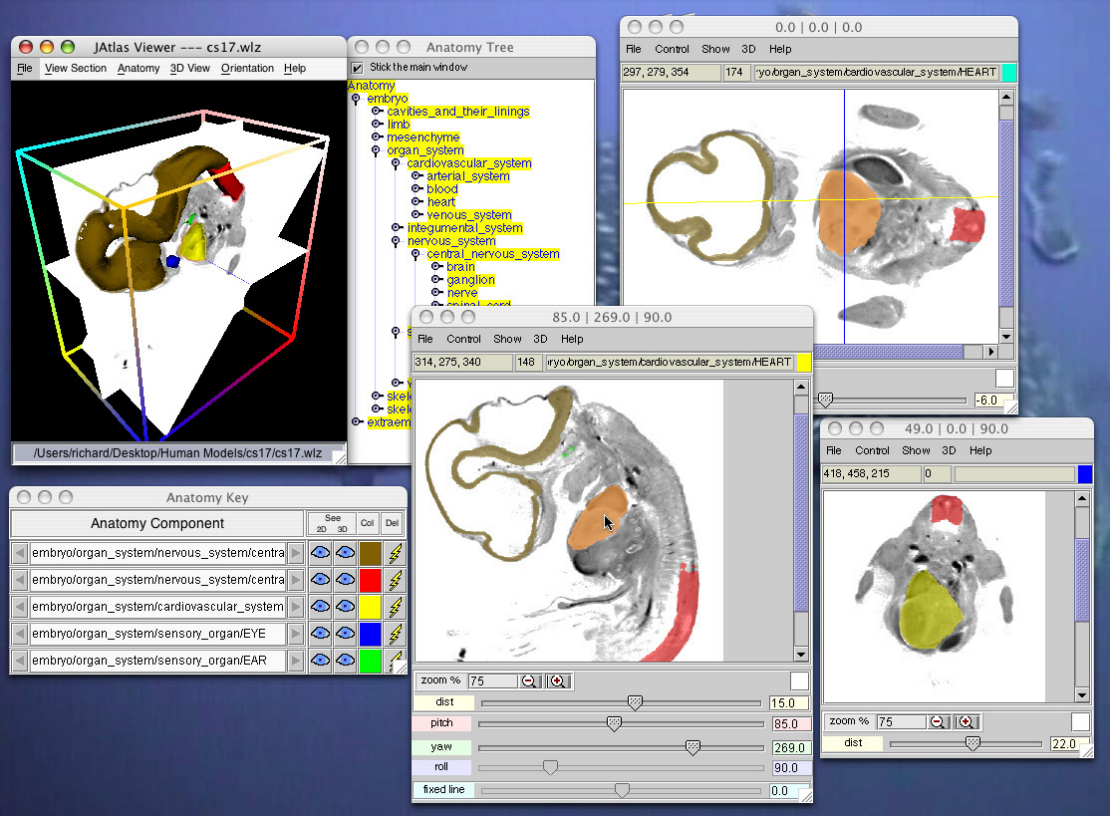
**JAtlasViewer interface. **Screen capture of the JAtlasViewer interface. Top-left: main window with 3D visualisation and anatomy tree; RHS: three section views through the data with some high-lighted anatomy; Bottom-left: current list of imported anatomy.

The user interface, shown in figure 1, has a primary window for the 3D view and top-level menu options, and a number of section-views for visualising the virtual sections cut through the data. The basic functionality of the viewer is to allow interactive digital resectioning of a 3D grey-level or voxel image. The special feature of this viewer is that any number of section views, each with an independent and arbitrary orientation and position can be displayed. To aid navigation through the volume a 3D feedback window is provided. This displays the bounding box of the 3D volume and a transparent surface, of e.g. the embryo model. In addition feedback of the current section position is provided in a number of selectable options: an intersecting polygon of the plane with the bounding box, display of the plane filled with a solid colour and display of the image of the section mapped onto the plane in the 3D view.

With appropriate data, the JAtlasViewer will import a mapped "anatomy". This is in two parts, a hierarchy of terms and a set of "domains" linked to specific terms in the hierarchy. The domains are 3D binary images which identify the region of space or set of voxels within the grey-level image associated with that term. The anatomy will then be used to provide feedback within the section views. These anatomy options, the controls for the section views and the main window options are discussed below.

### Main dialog

When the application is invoked a top-level dialog is presented to the user. Before anything can be displayed the user must select a grey-level 3D image. Currently this must be formatted as a Woolz image, but converters for many 3D formats are available from the EMAP web site. Once read in, the bounding box of the 3D voxel image will be displayed in the main window along with a surface representation of the data if available. This 3D view can be manipulated interactively using the cursor to provide views from arbitrary orientations and positions.

The menu options of the primary window are:

#### File

Commands to open image data, save views, save and restore settings, recent file-list and quit.

#### View Section

Select a section view through the voxel model. A new window will display one of the pre-set sections which are transverse, frontal and sagittal planes if the image model is appropriately aligned. Each of these can be set to display views at arbitrary orientations and locations within the 3D volume image.

#### Anatomy

If the voxel model data is configured with a set of anatomical regions, these can be selected from the menu and displayed in the section views. For the EADHB and EMAP atlases the menu hierarchy corresponds to the HUMAT and EMAP anatomy ontologies.

#### 3D View

Options to control the 3D visualisation in the main window, toggle the visibility of 3D surface, bounding box and intersection lines, display the focus section and selected anatomy.

#### Orientation

Preset 3D orientations to provide standard viewing directions.

#### Help

On-line help menu.

The 3D view window displays the bounding box of the opened volume and a transparent view of the embryo surface. This surface is pre-determined and stored in the visualization toolkit (VTK[19]) format. The 3D rendering is programmed in Java 3D, the objects (surface and bounding box) inside the 3D view window can be freely interactively manipulated with controls (using button drag) for rotation, translations and zoom (translation towards the viewer).

If an anatomy hierarchy and associated data files are provided then an additional window will allow browsing through the ontology and selection of components for display both in the section views and the 3D view window. As for the embryo surface, the surface models are pre-calculated and stored in VTK format. The data layout recognized by the JAtlasViewer is described in detail on the EMAP web-site.

### Section views

Each Section View is displayed in its own Section Viewer, either inside the main window (Microsoft Windows style) or in an independent external window. Section Viewers are Java components that can be easily imported into other applications. The primary viewing control is to move the view plane-parallel through the image volume as a form of "digital microtome" with section thickness determined by the underlying resolution of the 3D image, i.e. moving the microtome by a single step will move to the next voxel in the stack. The assumption is that once the section orientation has been determined the typical use will be to explore the volume in this fashion. The section position is determined by the "distance" parameter which is the voxel distance from the fixed point (by default in the centre of the bounding-box). Section orientation is selected by setting a number of view-angles. These control the view-direction which is perpendicular to the view-plane. We use the standard viewing angles defined by [20] which are related to the Euler angles of rotation [21]. Two of the angles determine the view-direction and the third is rotation around that direction. These angles can be understood in nautical/aeronautical terms as *pitch, yaw *and *roll *respectively. These viewing controls are hidden by default.

In addition to the primary view-direction controls there are options to assist navigation. These are **View-mode: **options for automatic roll determination in terms of the pitch and yaw values.

**Fixed-point: **select the fixed point used as the centre of rotation. The effect of setting this is to keep that voxel in view for all view-directions provided the "distance" is zero.

**Fixed-line: **set a second fixed-point and constrain the view so that both fixed points remain in the section. The effect of this is to reduce the degrees of freedom to a single parameter of rotation around the line between the two points.

The remaining controls for each section view are to set the feedback options including between section views, between the section view and the 3D view and to allow saving of the view and its settings (view parameters). The within view and between views feedback options are provided by the "Show" menu. This provides toggle controls to enable:

• cursor position in the reference image coordinate space and image grey-value to be displayed,

• line of intersection with section views. If two views intersect then the line of intersection is displayed in the appropriate colour,

• anatomy feedback – shows the domain and name of the anatomical component under the current cursor position,

• visible fixed point,

• visible fixed line.

The 3D view can provide feedback of the viewed sections in terms of the 3D volume. For most users these are important aids to understanding the position and direction of the viewed section. Most publications adhere to a convention for displaying section images, with this interface it is possible to view section data at any orientation and direction, i.e. depending on the view-direction the section may appear "reversed", so positional and directional feedback is critical. The positional feedback is provided in a number of forms but all indicate the intersection of the viewed plane with the bounding-box of the reference image. The most informative choice is to use texture mapping to render the grey-level image of the section into the 3D view. This is computationally expensive and so two other options are provided. These use the intersection polygon between the section plane and bounding box, either as-is, or filled with solid colour. The directional feedback is optional and provided by an arrow displayed in the 3D view.

### Anatomy manager

**Figure 2:**
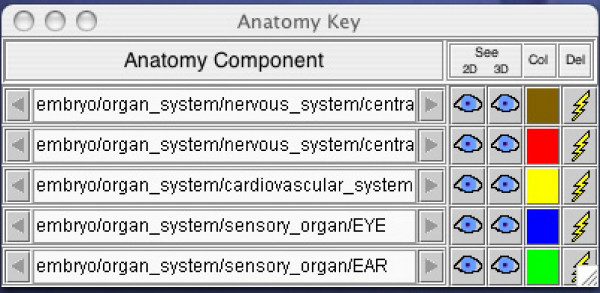
**Anatomy manager interface. **Anatomy key interface to control displayed colour, anatomy component name and visibilility in the 2D and 3D views.

The primary purpose of the JAtlasViewer is to provide an integrated viewer for 3D *atlases*. These comprise a grey-level (or potentially colour) reference image and a set of domains or regions which are associated with terms in a text hierarchy. For a geographic atlas these would correspond to the physical geography and the areas associated with individual countries. The hierarchy would then list the country names, perhaps under continents and split into counties. For EADHB and EMAP the reference image is the voxel reconstruction of the embryo and the domains are delineated anatomical components. The hierarchy of terms are the corresponding anatomy ontologies [22,23]. The user can select anatomical terms from the ontology for display in the section and 3D views. Once selected the component is handled by the *Anatomy Manager *(see fig 2) which controls the display properties visibility and colour. The anatomy-manager displays the full component name, visibility control toggles, colour chooser and a delete button. This style has been adopted because the number of possible component selections is large (15–500 depending on stage) and thus the user requires detailed control. In addition, although only selected terms in the anatomical hiearchy have corresponding domains defined, combinations of domains are generated "on-the-fly" so that larger scale structures can also be visualized.

The **colour chooser button **allows the user to change the colour of an anatomy component using a standard color chooser dialogue. The change is reflected immediately in all open Section Views and and the 3D feedback window..

The **text field **displays the full name of the anatomy component. Anatomy components fall into 2 broad hierarchies starting either at *embryo *or *extra-embryonic component*. The intervening higher level structures are separated with "/" (slash) and the final part of the name is capitalised. An asterisk following a name indicates that this is an atomic component, referred to in the anatomy menu as a (*domain*). Anatomical components are selected from the anatomy menu using a left mouse click. Higher level components or structures may also be selected from the anatomy menu using a right mouse click or a combination of the *shift *key and (left) mouse click. The anatomy name may be scrolled by dragging the mouse left or right inside the text box.

The visibility toggles select whether a component is displayed in the section views and in the 3D feedback window. This fine control helps the process of analysis and allows the user to build up a visualization showing all or parts of the anatomy. The **delete **is a toggle control which removes an anatomy component from the table.

## Conclusion

3D images are in widespread use in medical and biological research and there are a large number of options to view this data, but many of these are commercial and expensive, and are architecture and operating system dependent. More recently atlases and spatially mapped databases in biomedicine have been developed and whilst these packages can provide solutions for browsing this data we believe a simple, free-to-use, open-source and architecture-neutral solution provides a useful tool for biological research and teaching. The JAtlasViewer is intended to fill this requirement. The viewer provides the browsing functionality to locate and display arbitrary sections through the data with simultaneous 3D display. The JAtlasVIewer can also read and display a full anatomy atlas.

The JAtlasViewer is programmed in Java. The 3D programming technology is Java3D, which is a wrapper to the OpenGL or DirectX libraries. The Java and Java3D runtime environment are freely available from the Sun Microsystems web site and in most systems Java is pre-installed. These techniques minimize the coding work and developing time. The file size of the JAtlasViewer is less than 1.5 MB. Java WebStart manages the deployment, installation, upgrade and launch via a simple click on a html page link or an icon in the WebStart application. It is portable to any operating system to which Java has been ported and is currently available for Windows, Linux, Solaris and Mac OS.

The JAtlasViewer design is of reusable and extensible components. Based on the viewer a 3D tie-point collector for capturing 3D to 3D correspondences, and an atlas viewer that can also import gene expression data, have been developed.

## Availability and requirements

• Project name: The Mouse Atlas Project

• Project home page: 

• Application download: 

• Operating system(s): Solaris, Linux, Mac OSX, MS Windows.

• Programming language: Java, ANSI C.

• Other requirements: Java 1.4, JavaDoc, Java 3D.

• License: GNU GPL

• Any restrictions to use by non-academics: None

## Authors' contributions

Authors GF and NB undertook the main Java development and implementation, BH and RB develop and maintain the Woolz image processing library and BH implemented the automatic generation of the JNI. DD, JK, MS and SL all contributed to the design and testing of the interface and the preparation of the Atlas data for use with the tool.
